# Exchange of High-Frequency Medical Monitoring Data Using Fast Healthcare Interoperability Resources for Interoperable Integration Into Clinical Practice and Research: Methodological Development Study

**DOI:** 10.2196/94282

**Published:** 2026-11-02

**Authors:** Sara Erma Kaya, Yutong Wen, Cynthia Sabrina Schmidt, Sina Warmer, Henning Schäfer, Vin Yeang Choo, Obioma Pelka, Jendrik Richter, Philipp Goos, Karsten Schmidt, Dagmar Krefting, Sven Zenker, Felix Erdfelder, Nora Bruns, Katarzyna Borys, Sylvia Thun, Felix Nensa, Kamyar Arzideh, René Hosch

**Affiliations:** 1 Institute for Artificial Intelligence in Medicine (IKIM), University Hospital Essen, Essen, Germany; 2 Institute of Diagnostic and Interventional Radiology and Neuroradiology, University Hospital Essen, Hufelandstraße 55, Essen, 45147, Germany, 49 20172377817; 3 Data Integration Center, Central IT Department, University Hospital Essen, Essen, Germany; 4 Institute for Transfusion Medicine, University Hospital Essen, Essen, Germany; 5 Center of Sleep and Telemedicine, University Hospital Essen - Ruhrlandklinik, Essen, Germany; 6 Department of Medical Informatics, University Medical Center Göttingen, Göttingen, Germany; 7 Institute of Medical Informatics, Heidelberg University, Heidelberg, Germany; 8 Department of Anesthesiology and Intensive Care Medicine, University Hospital Essen, University Duisburg-Essen, Essen, Germany; 9 Applied Medical Informatics (AMI) Lab, Institute for Medical Biometry, Informatics, and Epidemiology, University Hospital Bonn, Bonn, Germany; 10 Applied Mathematical Physiology (AMP) Lab, Department of Anesthesiology and Intensive Care Medicine, University Hospital Bonn, Bonn, Germany; 11 Staff Unit for Medical & Scientific Technology Development & Coordination (MWTek), University Hospital Bonn, Bonn, Germany; 12 Department of Pediatrics I, University Hospital Essen, Essen, Germany; 13 Berlin Institute of Health (BIH) at Charité - University Hospital Berlin, Berlin, Germany

**Keywords:** Health Level 7 Fast Healthcare Interoperability Resources, time series, interoperability, medical waveform data, HL7 FHIR profiles, digital health

## Abstract

**Background:**

Continuous patient monitoring generates vast volumes of high-frequency monitoring data, such as electrocardiogram, electroencephalography, and invasive blood pressure tracings, that are often persisted in heterogeneous, noninteroperable formats, limiting integration with electronic health records and secondary use. Although Fast Healthcare Interoperability Resources (FHIR) supports general health data exchange, native support for high-frequency medical monitoring data, including associated metadata and data quality metrics, remains limited.

**Objective:**

This study aims to develop a modality-agnostic FHIR package implementing a *Metadata-Proxy* pattern to enable scalable, semantically precise representation of high-frequency medical monitoring data, decoupling structured clinical metadata from raw signal storage.

**Methods:**

This study provides the FHIR package Time Series Integration and Data in Endpoints (TIDE), a collection of FHIR Release 4 artifacts for interoperable exchange and the standardization of high-frequency monitoring clinical data, using electroencephalography as the primary use case while remaining applicable to generic medical monitoring data. The core TIDE *Observation* profile separates technical metadata and analytical summaries stored directly in FHIR from high-volume raw data referenced via *Endpoint* resources. Quantitative results are standardized with pattern slicing, including sampling frequency, statistical summaries, and data quality metrics. Semantic interoperability was achieved through a hybrid terminology binding, using Systematized Nomenclature of Medicine Clinical Terms, Logical Observation Identifiers Names and Codes for modalities, and custom *CodeSystems* for technical metadata, waveform modalities, channel identifiers, and connection types not covered by standard terminologies. All profiles were authored in FHIR Shorthand and compiled with SUSHI to produce a validated Implementation Guide conformant with the FHIR Release 4 core specification.

**Results:**

The resulting framework provides a modality-agnostic structure for high-frequency monitoring data, comprising 7 primary FHIR artifacts that define 16 distinct component slices for metadata and signal analysis results. *Observation* resources reference associated *Device* and *Endpoint* instances, encapsulate acquisition parameters, and link to full-time-series repositories. Controlled vocabularies ensure unambiguous exchange, thereby enabling downstream use in research and decision support. The architecture decouples the metadata layer from the storage layer via a standardized *Endpoint* extension, enabling efficient querying of signals without retrieving large amounts of raw high-frequency monitoring data.

**Conclusions:**

TIDE provides a reusable, standards-compliant framework for integrating continuous high-frequency monitoring data into FHIR-based ecosystems. By separating semantically rich, queryable metadata from raw signal storage, TIDE enables longitudinal patient monitoring and automated signal quality-based triage without requiring modification of existing signal storage infrastructure.

## Introduction

Continuous physiological monitoring plays a critical role in modern health care, enabling timely detection of clinical changes and supporting informed therapeutic decisions. Technologies such as electroencephalography (EEG), electrocardiography (ECG), and invasive blood pressure monitoring generate large volumes of high-frequency time series data, capturing subtle physiological dynamics over extended periods [1]. These data can provide valuable insights into patient status, particularly in intensive care [2-4], neurology [5-7], and cardiorespiratory monitoring [8].

Despite their potential, most high-frequency monitoring data are not directly integrated into electronic health records but instead are typically stored only temporarily within vendor-specific data warehouse solutions (eg, Hadoop-based systems) [9]. While the de facto standard European Data Format (EDF) is often implemented as an export format [10], many vendors use proprietary formats or open standards such as the common open standard “Digital Imaging and Communications in Medicine,” often supplemented with vendor-specific private tags. Digital Imaging and Communications in Medicine, originally defined for medical imaging and supported by Picture Archive and Communication Systems, has been expanded to represent waveforms and long-term biosignal recordings [11-13]. However, in such settings, raw waveform data are frequently treated as static payloads and lack standardized, interoperable representations, which prevent seamless exchange across systems and institutions and limit opportunities for secondary use in research and clinical decision improvement [14].

At the same time, Fast Healthcare Interoperability Resources (FHIR) [15] has emerged as one of the most widely adopted standards for representing and exchanging health care data [16]. By defining modular resources and enabling their adaptation through profiling, FHIR facilitates the creation of use case–specific data structures while maintaining compatibility with broader interoperability frameworks [17]. However, native support for continuous, high-frequency monitoring data has not yet been comprehensively established [1,11]. Although waveform snippets are technically permitted, long-duration, high-volume recordings characteristic of continuous monitoring do not align well with current FHIR usage patterns, leading to resource inflation and inefficient data retrieval [1]. This limitation becomes particularly evident in multicenter research infrastructures such as ACRIBiS [18], a consortium project integrating biosignal-derived phenotypes into interoperable cardiovascular risk assessment workflows. Addressing this limitation requires an architectural shift in which FHIR serves as a metadata layer while efficiently handling raw data payloads. Such integration could facilitate longitudinal patient monitoring across care settings, enable multicenter data aggregation, and provide a foundation for advanced analytics and clinical decision support systems operating on interoperable data.

To achieve this, specialized FHIR profiles are required to extend existing resources with structures that can represent high-frequency measurements, link them to acquisition devices, and reference complete time series stored in external repositories. Profiles must also ensure semantic interoperability by using controlled vocabularies, such as Systematized Nomenclature of Medicine Clinical Terms (SNOMED CT) [19,20] or Logical Observation Identifiers Names and Codes (LOINC) [21,22], and by defining purpose-built *CodeSystems* to provide canonical, machine-readable definitions for technical signal-processing concepts that are not yet represented in existing international vocabularies, while remaining aligned with FHIR’s extensibility principles to facilitate future harmonization.

This paper presents the design and implementation of Time Series Integration and Data in Endpoints (TIDE), a foundational, modality-agnostic FHIR framework for the interoperable representation of continuous medical monitoring data. The core of the proposed framework is the TIDE *Observation* profile, which enforces a structured representation for technical metadata and derived analytics while referencing high-volume raw data in specialized external repositories via a *Metadata-Proxy* pattern. To assess the viability and flexibility of this approach, EEG is used as an illustrative example of a complex, high-frequency monitoring scenario, while the framework remains applicable to continuous medical monitoring data across modalities. This work establishes a reusable, standards-compliant pattern for integrating diverse continuous monitoring modalities into the FHIR ecosystem.

## Methods

### Background

FHIR supports interoperable data exchange through standardized resources, Representational State Transfer Application Programming Interface (RESTful API) [23], and serialization formats such as JSON and XML [24-26]. Clinical and technical information is represented using standardized resources, such as *Observation* for clinical measurements, *Device* for recording equipment, and *Endpoint* for referencing external services and data repositories [27]—the latter forming the architectural foundation for TIDE’s storage-agnostic design. Although the *SampledData* data type permits embedding sequential measurement values within an *Observation* resource, this approach requires complete deserialization prior to analysis and does not support direct integration with time series–optimized storage backends, motivating an alternative architectural approach for high-frequency monitoring data [1,14].

To support specialized use cases, FHIR allows for the creation of profiles to constrain or extend base resources to meet domain-specific requirements while maintaining interoperability. In this study, the TIDE profiles were developed based on FHIR Release 4 (R4), authored in FHIR Shorthand [28], and compiled with the SUSHI reference implementation (version 3.17.0) [29]. The resulting artifacts were subsequently processed by the Health Level 7 (HL7) FHIR Implementation Guide Publisher (version 2.3.4), which invoked the official HL7 FHIR validator against the FHIR R4 (version 4.0.1) core specification to produce a conformant Implementation Guide.

### Study Design and Analytical Approach

This study employed a use case–driven design approach to develop FHIR-compliant artifacts for representing continuous medical monitoring data, progressing from use-case selection and requirements analysis to profile modeling and implementation. Multichannel EEG was used as the primary use case, representing a complex, data-intensive monitoring scenario that required a structured representation of both acquisition parameters and derived analytical metrics, including statistical signal summaries, signal stability indices [30], and anomaly detection flags. Due to its high sampling rates, multiple simultaneous channels, and dual technical and clinical quality descriptors, EEG served as a rigorous stress test for a modality-agnostic framework.

Within the scope of this work, we assume that continuous monitoring data are incrementally represented as FHIR resources during ongoing monitoring. This assumption informed a segmentation strategy in which each preexisting, time-bounded recording interval (eg, an EDF run) is published as a self-contained *Observation* resource with fixed temporal boundaries, avoiding open-ended time periods while maintaining auditability and traceability through standard FHIR resource versioning. For a flat data model, where signal data were segmented into the full recording window per run, each interval within a monitoring session is assigned a *preliminary* status except for the last, which is assigned a final status once the session concludes. In the hierarchical model (see Multimedia Appendix 1), used when all channels of a recording are stored in a single EDF file, all *Observations* are currently published with a final status. Algorithmic splitting of a continuous stream into fixed windows and handling of overlapping, duplicated, or late-arriving boundaries remain outside the scope of this proof-of-concept (PoC). Although the evaluation was limited to EEG to ensure focus and reproducibility, the approach was designed to apply to other continuous monitoring scenarios.

To illustrate practical applicability, the framework was positioned within a conceptual end-to-end data analytics pipeline, spanning raw monitoring data collection, signal processing, and FHIR server storage of structured metadata and repository references via the TIDE *Observation* profile, as illustrated in Figure 1. The pipeline serves as an architectural reference demonstrating how TIDE artifacts would be instantiated in a real-world integration scenario.

**Figure 1.**
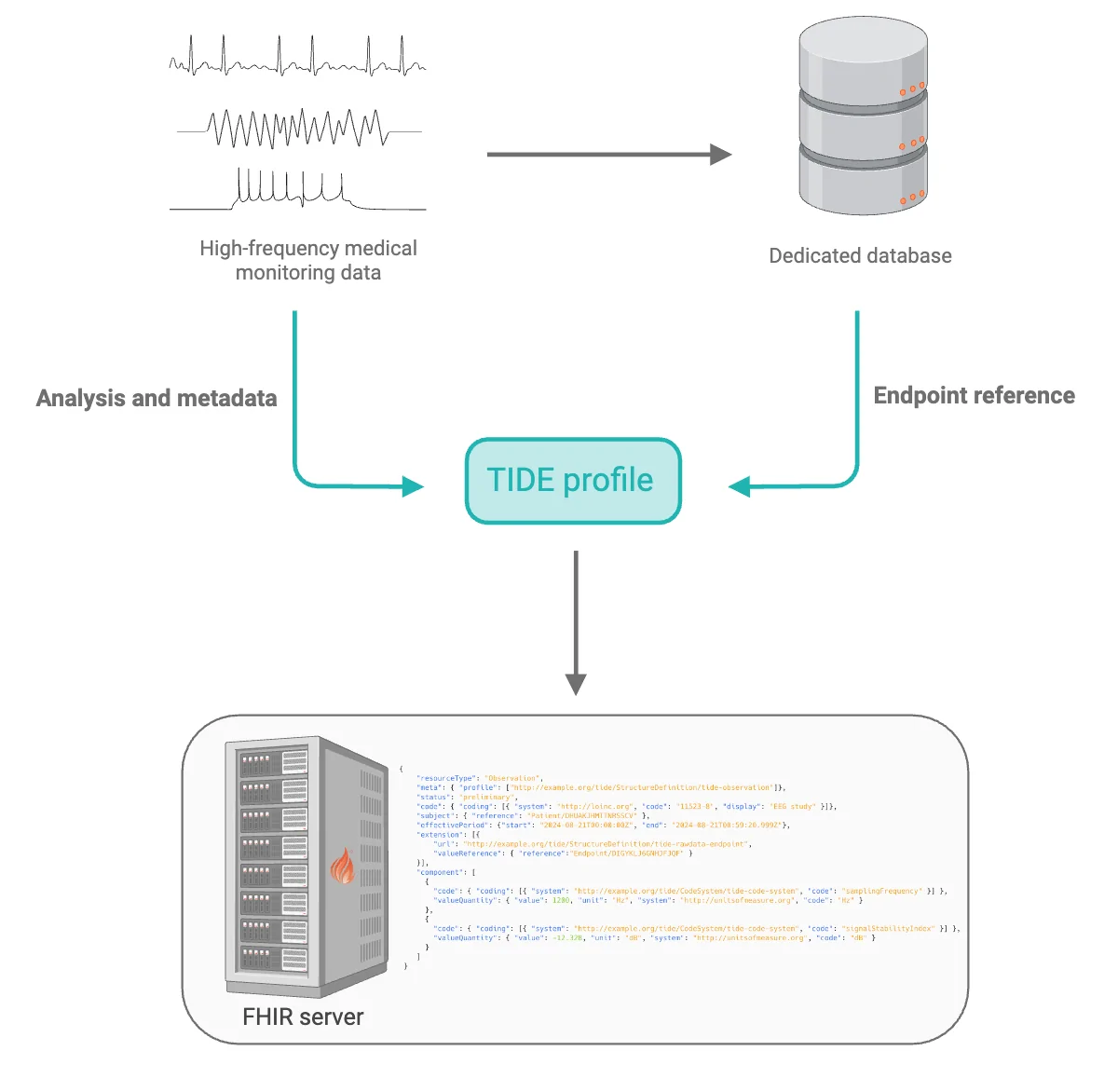
Overview of technical workflow and HL7 FHIR resource instantiation. The pipeline illustrates the transformation of raw physiological signal data (eg, EEG, ECG, and EMG) into a standardized TIDE *Observation* resource. The integrated JSON snippet demonstrates the implementation of the *Metadata-Proxy* pattern, storing acquisition parameters and signal quality metrics directly within the FHIR infrastructure. Created with BioRender [31]. ECG: electrocardiogram; EEG: electroencephalogram; EMG: electromyogram; FHIR: Fast Healthcare Interoperability Resources; HL7: Health Level 7; TIDE: Time Series Integration and Data in Endpoints.

### Ethical Considerations

This methodological development study used 2 publicly available, deidentified EEG datasets from OpenNeuro [32,33], which were used solely as test data to demonstrate the functionality of the developed TIDE FHIR package. No participants were recruited or contacted, no new human subject data were collected, and the investigators did not access identifiable personal information. The Ethics Committee of the Medical Faculty of the University of Duisburg-Essen reviewed the study and confirmed that no ethics review or informed consent was required, as this research project involves neither an intervention into a person’s psychological or physical integrity nor the use of biological materials or data that can be linked to a specific individual (reference number 26‐13359-NBO).

### Profile Structure

The proposed profile architecture consisted of a central TIDE *Observation* resource with multiple relationship types to referenced resources, *extensions*, and *component* elements. In this architecture, the FHIR resource served as a semantically rich index and metadata container. At the same time, high-volume raw monitoring data were offloaded to specialized external repositories, such as databases or cloud-native object storage, selected according to institutional requirements, preserving the full fidelity of the raw signal data within purpose-built storage systems accessible via standardized *Endpoint* references. The overall structure of the TIDE framework is shown in Figure 2.

**Figure 2.**
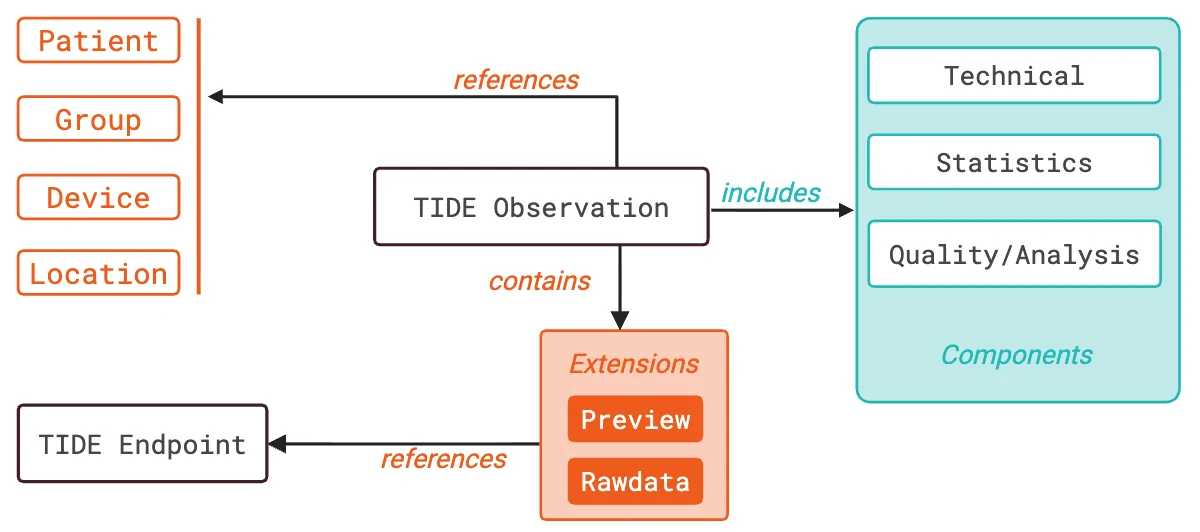
TIDE framework structure. This diagram illustrates the core structure and relationships of the TIDE *Observation* profile, designed to standardize high-frequency medical monitoring data. It demonstrates the main *Observation* profile and its association with referenced resources, the TIDE *Endpoint* profile, statistical component blocks, and two custom extensions (*RawDataEndpoint* and *PreviewEndpoint*). For clarity, the optional hierarchical model, in which a parent TIDE *Observation* references per-channel *TIDEObservationChild* resources via *hasMember,* is omitted here and shown in Figure 3 and Multimedia Appendix 1. Image created with BioRender [34]. TIDE: Time Series Integration and Data in Endpoints.

The framework’s architectural core is the TIDE *Observation*. Unlike traditional implementations that use the *SampledData* data type [1,14,27], TIDE implements the *Metadata-Proxy* pattern. In this pattern, the *Observation* resource serves solely as a searchable index of technical metadata, quality metrics, and references to storage locations, rather than storing the bulk data itself. The profile implements open pattern slicing to capture analytical insights, allowing additional, unspecified component slices beyond those explicitly defined—enabling modality-specific extensions without violating profile conformance, categorized into 3 domains: acquisition parameters, statistical signal summaries, and data quality metrics. Additionally, the profile incorporates 2 custom extension mechanisms: the mandatory *RawDataEndpoint* extension, establishing the primary link to the raw signal repository, and the optional *PreviewEndpoint* extension, enabling lightweight signal visualization without full data retrieval—each referencing a dedicated TIDE *Endpoint* profile to bridge the gap between the FHIR metadata layer and the raw data persistence layer. The referenced raw data payloads remain outside the FHIR system and are not intended to be managed as FHIR resources. To accommodate both compact single-resource scenarios and per-channel granularity in multielectrode recordings, the framework supports 2 complementary *Observation* modeling patterns. In the flat single-resource model, all 16 component slices—covering global acquisition parameters, statistical signal summaries, and data quality metrics—are encoded as components of a single TIDE *Observation* resource. In the hierarchical structure, where a single recording contains multiple channels, a parent *Observation* captures global metadata via its component slices and references individual per-channel child *Observations* through the standard FHIR *hasMember* element, making each channel’s signal metrics independently searchable via the FHIR RESTful API without requiring retrieval of the full multichannel resource. A detailed channel reference with per-electrode binding examples for the hierarchical model is provided in Multimedia Appendix 1.

To ensure clinical perspectives, the profiles were reviewed by medical experts from several fields, including neuroradiology, radiology, transfusion medicine, sleep medicine, pediatrics, and intensive care medicine, to validate the representational adequacy of the defined requirements.

**Figure 3.**
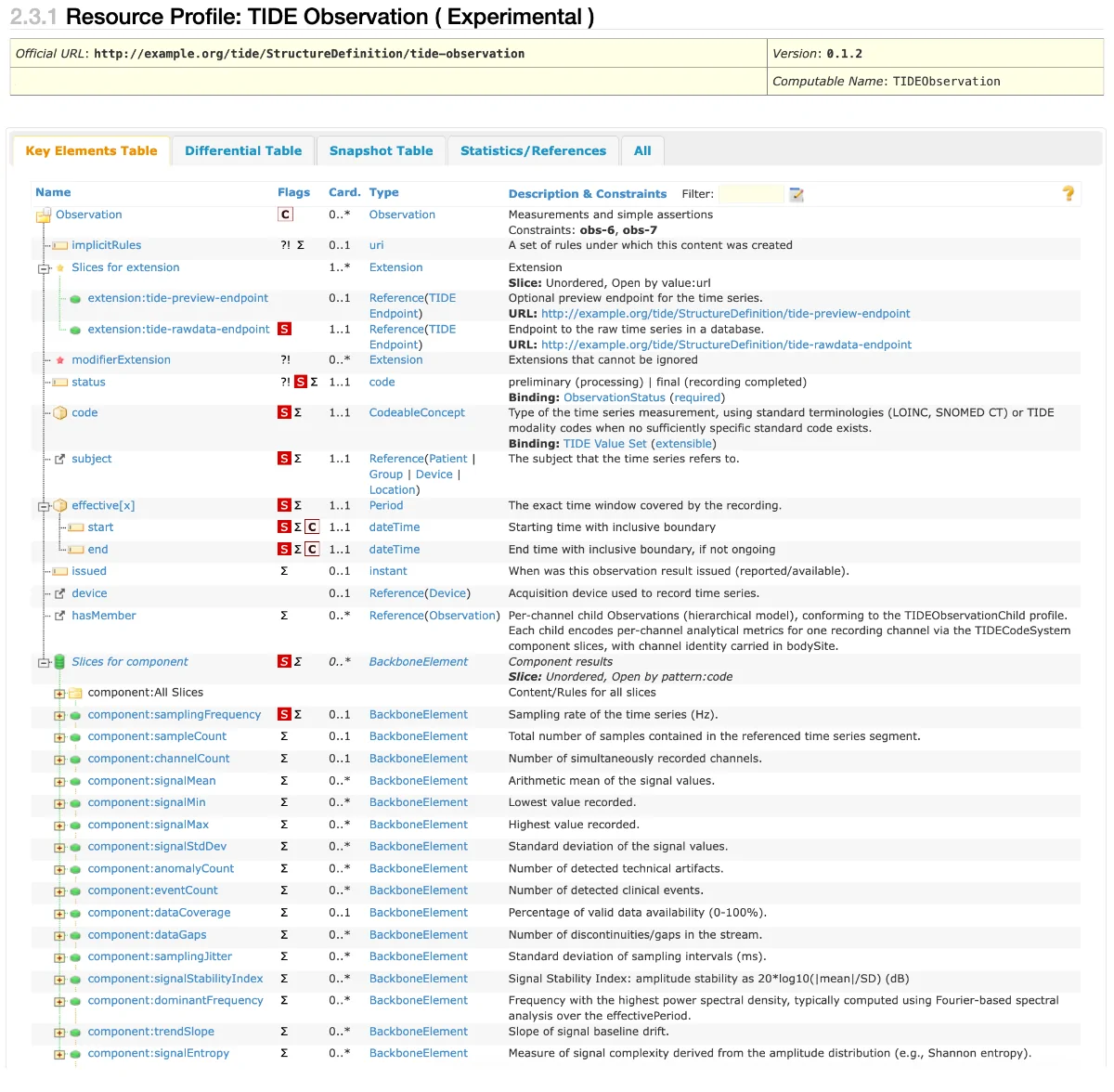
Overview of the TIDE *Observation* profile. This visualization illustrates the key components and constraints of the proposed profile, including core metadata, endpoint extensions, the 16 analytical component slices, and *hasMember* references to per-channel child *Observations* supporting the hierarchical model. TIDE: Time Series Integration and Data in Endpoints.

## Results

### General Overview

The proposed TIDE framework comprises 7 primary FHIR artifacts: the core TIDE *Observation* profile, the TIDE *Endpoint* profile, 2 custom extensions for external data linkage (*RawDataEndpoint* and *PreviewEndpoint*), 2 dedicated *CodeSystems* (*TIDECodeSystem* for technical metadata and signal quality descriptors, and *TIDESupplementalCodes* for waveform-specific modality codes), and 1 *ValueSet* (*TIDEValueSet*). Supporting artifacts for the hierarchical model (*TIDEObservationChild*, *TIDEChannelCodeSystem*, and *TIDEChannelValueSet*) are described in Multimedia Appendix 1; the *TIDEConnectionTypeCodes CodeSystem* used in the PoC deployment is described in Multimedia Appendix 2. To assess TIDE’s feasibility, a PoC implementation was performed using 2 publicly available EEG datasets, demonstrating both the flat single-*Observation* model and the hierarchical parent/child model using *hasMember*. Details of the datasets, implementation pipeline, quantitative evaluation, and example FHIR queries are presented in Multimedia Appendix 2.

### TIDE Observation

The TIDE *Observation* profile, depicted in Figure 3, was derived from the base FHIR *Observation* resource to support diverse monitoring contexts.

The *subject* reference was made mandatory (cardinality *1..1*) while retaining the reference types *Group*, *Device*, and *Location* alongside *Patient* resources, supporting diverse use cases ranging from clinical patient monitoring to technical device calibration. This ensures the potential inclusion of cohort studies, calibration data, or environmental monitoring data. The *effective[x]* element was constrained to *Period* to ensure temporal bounding and enable precise longitudinal querying. Both start and end timestamps from the *effective[x]* element are mandatory (cardinality *1..1*), ensuring that every published segment has explicit temporal boundaries.

Additionally, the profile defined 16 distinct *component slices* representing technical metadata (*samplingFrequency*, *sampleCount*, and *channelCount*), statistical summaries (*signalMean*, *signalMax*, *signalStdDev*, and *signalMin*), and data quality metrics. The data quality domain includes basic coverage and gap metrics (*dataCoverage*, *dataGaps*) alongside advanced signal-processing descriptors, including a signal stability index (*signalStabilityIndex*), sampling jitter (*samplingJitter*), dominant frequency (*dominantFrequency*), trend slope (*trendSlope*), and signal entropy (*signalEntropy*), enabling automated, threshold-based signal quality filtering at the FHIR metadata level without requiring raw data retrieval. Critically, a semantic distinction was drawn between technical artifacts (*anomalyCount*) and clinically relevant physiological events (*eventCount*), supporting differential quality assessment. For channel-level metric binding, TIDE additionally supports a hierarchical parent-child *Observation* model in which per-channel child *Observations* (*TIDEObservationChild*) are identified via the structured *bodySite* element, bound extensibly to the *TIDEChannelValueSet* with codes from the *TIDEChannelCodeSystem,* as detailed in Multimedia Appendix 1.

### TIDE Endpoint

The TIDE *Endpoint*, depicted in Figure 4, was derived from the base FHIR *Endpoint* resource and provides a standardized mechanism for referencing high-frequency raw monitoring data stored in external repositories. Its primary purpose was to establish a consistent, interoperable means of referencing externally hosted payloads, enabling storage-agnostic access to heterogeneous signal repositories while preserving the data’s native representation.

**Figure 4.**
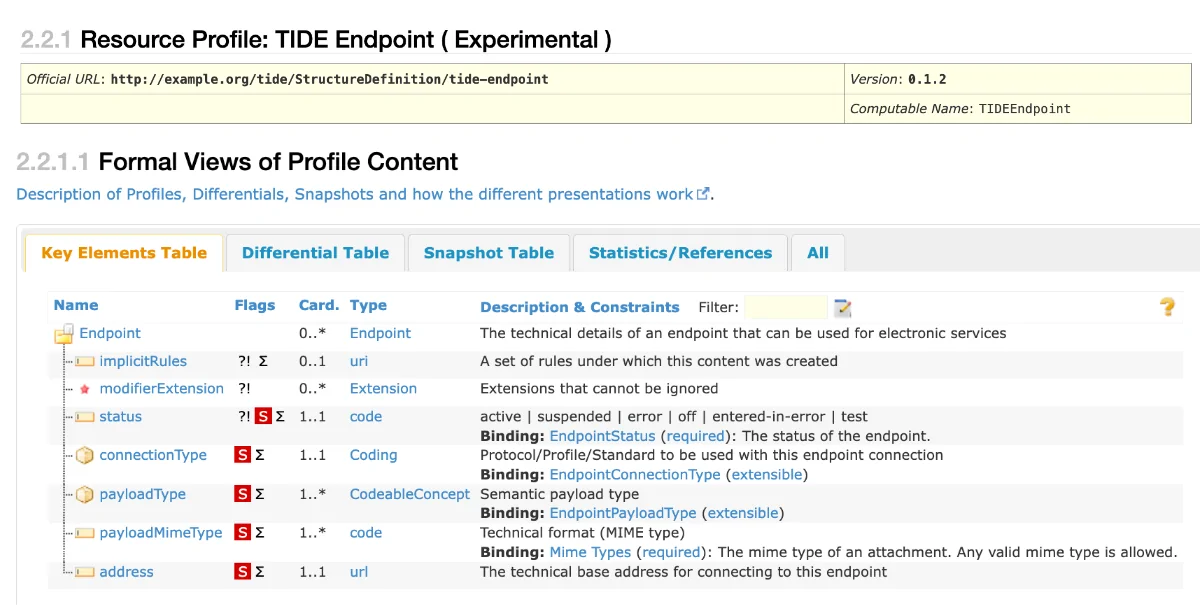
Overview of the TIDE *Endpoint* profile. This visualization illustrates the key elements and constraints of the TIDE *Endpoint* profile, including all mandatory elements and the inherited binding of payloadMimeType to the MimeTypes value set. The profile provides a standardized mechanism for referencing external high-frequency monitoring data repositories, enabling storage-agnostic integration within the TIDE framework. MIME: Multipurpose Internet Mail Extensions; TIDE: Time Series Integration and Data in Endpoints.

The profile defines 5 mandatory elements. Four of them (*connectionType*, *address*, *payloadMimeType,* and *payloadType*) collectively provide the information required to identify and access the endpoint without requiring out-of-band negotiation, while s*tatus* indicates the current operational state.

The *connectionType* element specifies the protocol or standard used for the connection, bound extensibly to the standard FHIR *EndpointConnectionType* value set. Because none of the standard codes adequately describes plain file retrieval over HTTPS, as used for the publicly hosted recordings in the PoC implementation, a supplemental *TIDEConnectionTypeCodes CodeSystem* was defined in Multimedia Appendix 2. The *address* element provides the concrete URL at which the endpoint can be reached. The *payloadMimeType* element ensures that consuming systems can parse the payload without prior negotiation. It retains the required binding to the MimeTypes value set inherited from the base FHIR R4 *Endpoint* resource, which admits any valid Multipurpose Internet Mail Extensions type. The *payloadType* element provides a semantic description of the payload content and is bound extensibly to the *EndpointPayloadType* value set, consistent with TIDE’s design principle of enforcing semantic precision at the core while accommodating institutional variability. As the PoC implementation used publicly accessible OpenNeuro datasets described in Multimedia Appendix 2, authentication and authorization for access-controlled deployments, payload versioning, and data-integrity verification are outside the scope of the current implementation.

While the profile does not further restrict *payloadMimeType* to a predefined set of formats, reflecting the absence of an established consensus on standard payload representations for high-frequency monitoring data, it requires the format to be explicitly declared. This design provides a concrete, forward-compatible extension point: as community consensus emerges, a recommended set of Multipurpose Internet Mail Extension types for specific monitoring modalities can be defined without breaking existing conformance.

This profile was referenced exclusively via the 2 custom extensions: the mandatory *RawDataEndpoint* extension and the optional *PreviewEndpoint* extension.

### Extensions

To bridge the gap between the FHIR metadata layer and the raw data persistence layer, 2 custom *extensions* were implemented, as illustrated in Figure 5. The mandatory *RawDataEndpoint* serves as the primary link to the payload. By referencing the TIDE *Endpoint* resource rather than a simple URL, the framework abstracted technical connection details, such as the connection protocol, payload format, and technical base address, from the *Observation*. This abstraction decoupled the clinical observation record from the underlying storage infrastructure: the backend repository can be replaced, migrated, or upgraded without requiring any modification to the FHIR *Observation* resources that reference it—a key property for long-term maintainability in evolving clinical IT environments. The optional *PreviewEndpoint* references a visualization middleware or thumbnail service, enabling client applications to render a lightweight visual preview of the signal without the network overhead of retrieving the full high-frequency dataset, allowing clinicians and researchers to rapidly assess signal characteristics before committing to full data retrieval.

**Figure 5.**
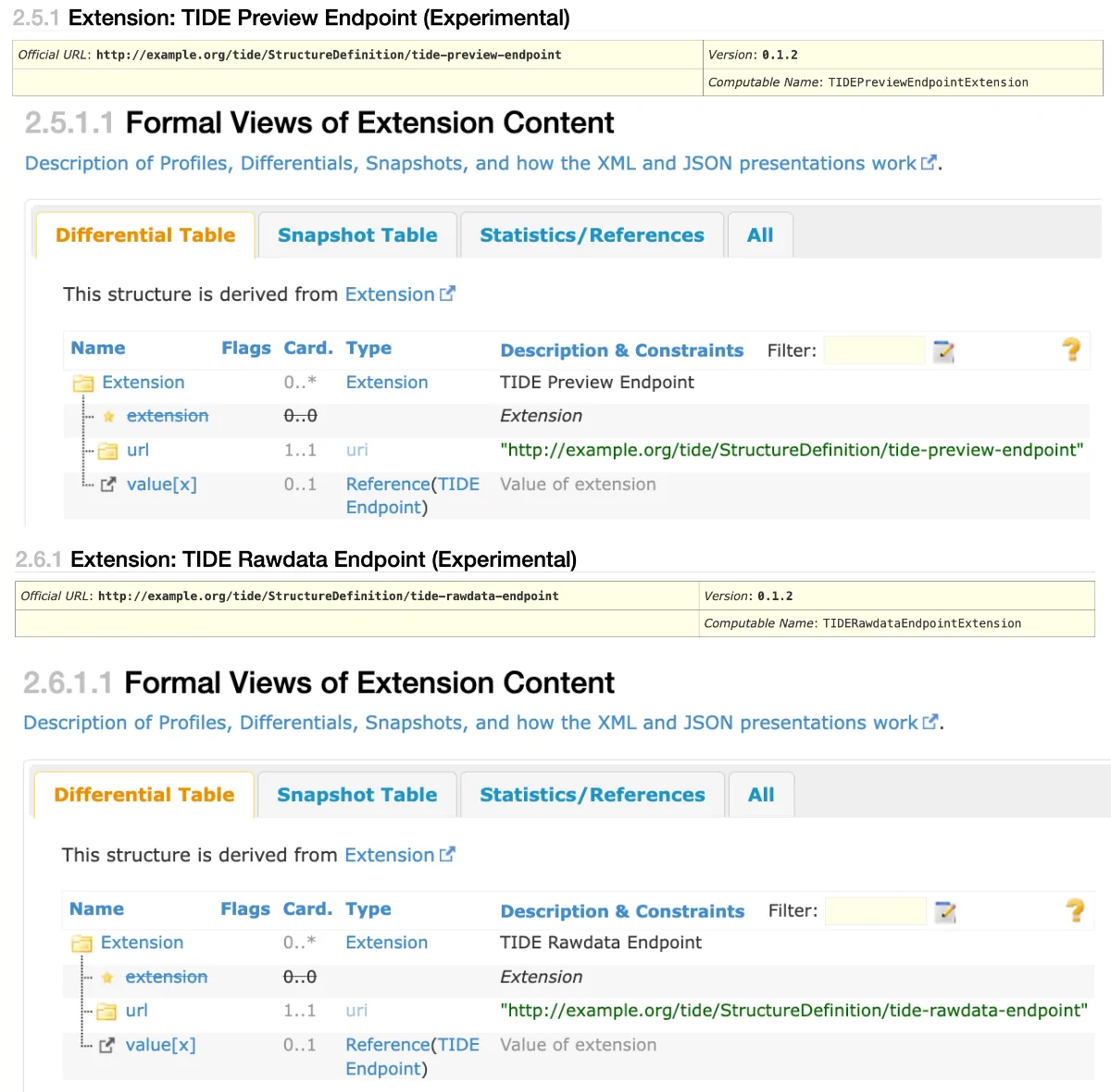
Overview of the *TIDE Preview Endpoint Extension* (top) and *TIDE Rawdata Endpoint Extension* (bottom). The *Extensions* enable structured references from FHIR *Observation* resources to external monitoring data services via standardized TIDE *Endpoint* resources. These endpoints specify technical access parameters, such as connection address and payload MIME type, enabling scalable storage, retrieval, and visualization of high-resolution waveform data outside the FHIR resource while preserving queryable metadata within FHIR. FHIR: Fast Healthcare Interoperability Resources; MIME: Multipurpose Internet Mail Extensions; TIDE: Time Series Integration and Data in Endpoints.

### Terminology

Existing standard terminologies provided insufficient coverage for 2 distinct requirements of the TIDE framework: technical signal-processing metadata, such as sampling jitter, signal entropy, and dominant frequency, which were not represented in LOINC or SNOMED CT; and high-resolution continuous waveform modality codes, for which standard terminologies lacked the specificity required to distinguish, for example, an invasive arterial blood pressure waveform from a derived blood pressure value. To address both gaps, 3 terminology artifacts were defined, as illustrated in Figures 6 and 7.

**Figure 6.**
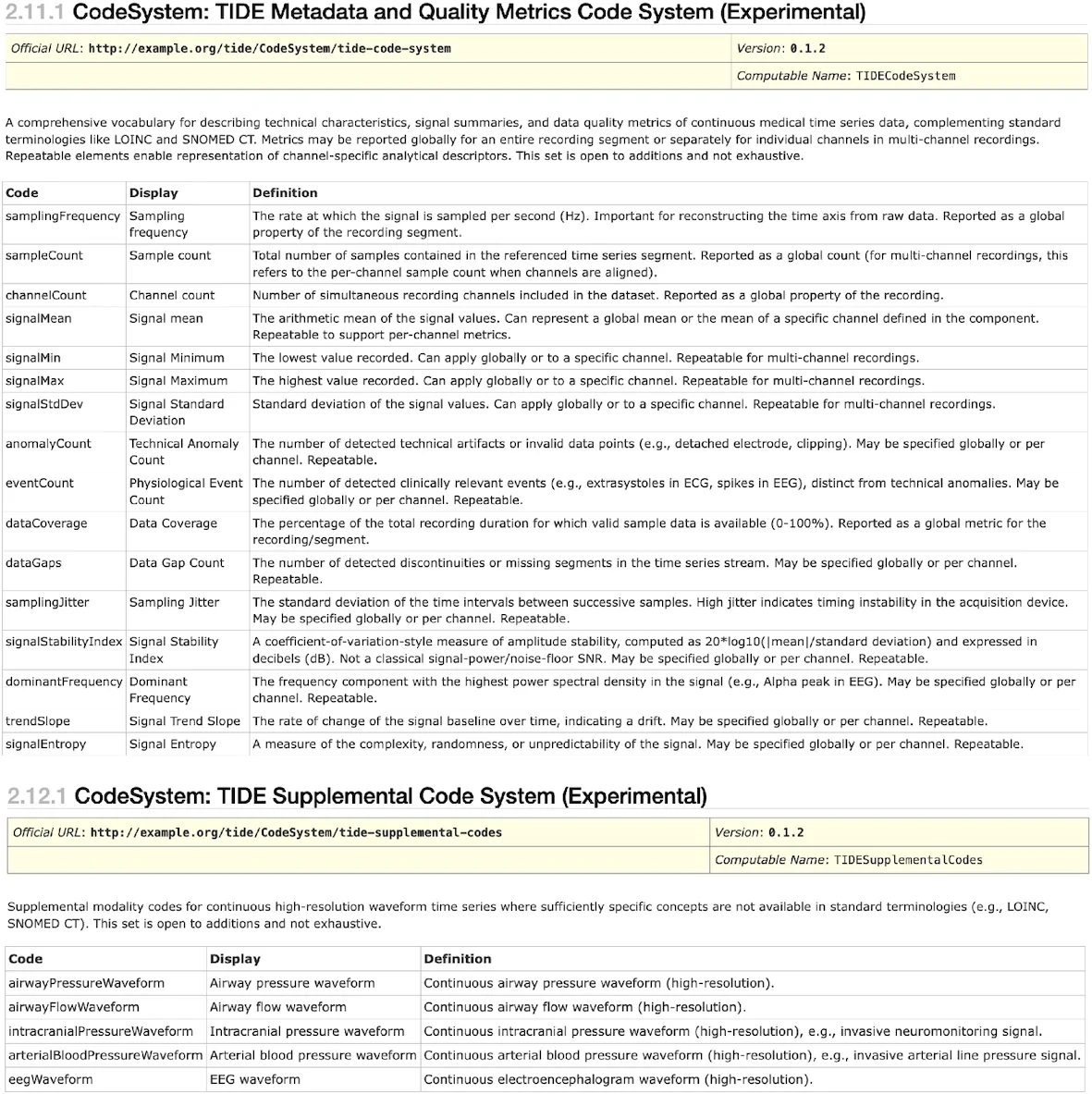
The *TIDECodeSystem* and *TIDESupplementalCodes CodeSystem*. The *TIDECodeSystem* provides canonical, machine-readable definitions for 16 technical signal-processing concepts spanning acquisition parameters (eg, *samplingFrequency*, *channelCount*), statistical signal descriptors (eg, *signalMean*, *signalStdDev*), and advanced quality metrics (eg, *samplingJitter*, *dominantFrequency*, *signalEntropy*) not covered by standard terminologies. The *TIDESupplementalCodes CodeSystem* provides 5 supplemental modality codes for continuous high-resolution waveform signals, including invasive arterial blood pressure and intracranial pressure waveforms, for which standard terminologies offer only derived or nonwaveform-specific codes. Both *CodeSystems* are open to additions and are not exhaustive. TIDE: Time Series Integration and Data in Endpoints.

**Figure 7.**
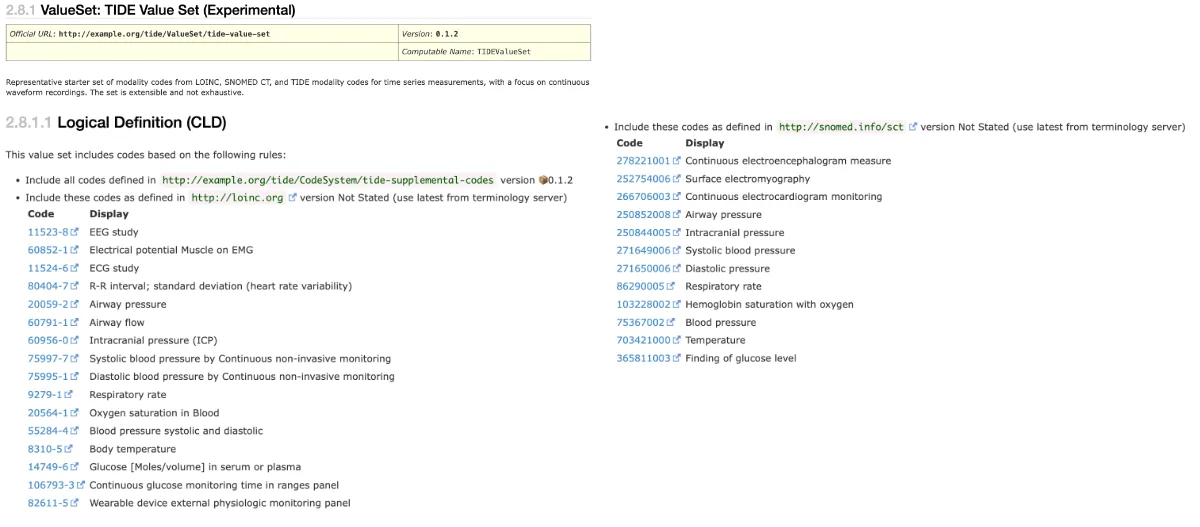
The *TIDEValueSet*. The *TIDEValueSet* aggregates modality codes from LOINC, SNOMED CT, and the *TIDESupplementalCodes CodeSystem* into a single extensible binding for the code element of the TIDE *Observation* profile. The included codes cover 6 clinical categories: neurophysiology, cardiology, pressure and flow monitoring, vital signs, metabolic parameters, and kinematics and wearables. The set is extensible and is not exhaustive, in alignment with FHIR’s extensibility principles. LOINC: Logical Observation Identifiers Names and Codes; SNOMED CT: Systematized Nomenclature of Medicine Clinical Terms; TIDE: Time Series Integration and Data in Endpoints.

The *TIDECodeSystem* defined canonical, machine-readable concepts for the 16 component slices of the TIDE *Observation*, covering acquisition parameters, statistical signal summaries, and advanced quality metrics that were not represented in existing international vocabularies. The *TIDESupplementalCodes CodeSystem* addresses a distinct gap at the modality level: while LOINC and SNOMED CT provided codes for derived physiological values such as systolic blood pressure, they lacked sufficiently specific codes for the corresponding continuous high-resolution waveform signals, such as invasive arterial line pressure tracings or intracranial pressure waveforms. Five such waveform-specific codes were defined in *TIDESupplementalCodes* as a structured extension point pending future harmonization with standard terminologies.

The *ValueSet* (*TIDEValueSet*) aggregated modality codes from LOINC, SNOMED CT, and the custom *TIDESupplementalCodes CodeSystem*, and the corresponding code element in the TIDE *Observation* was bound to this *ValueSet* to identify the recording’s modality. This extensible set was organized into domain-specific categories, including neurophysiology, cardiology, pressure and flow monitoring, vital signs, metabolic parameters, and kinematics and wearables. These categories reflected the primary clinical domains in which continuous high-frequency monitoring was currently employed and were selected to maximize coverage of realistic deployment scenarios while maintaining a manageable, curated starter set. Importantly, these definitions were not intended to be exhaustive. In alignment with FHIR’s extensibility principles, all terminology artifacts remain adaptable, allowing the inclusion of additional high-resolution modalities to support specific clinical scenarios as required.

## Discussion

### Principal Results

This study introduces TIDE, a modality-agnostic FHIR framework that enables the interoperable exchange of high-frequency medical monitoring data across health information systems. By implementing the *Metadata-Proxy* pattern, TIDE establishes a structured bridge between external signal repositories and the clinical interoperability layer, using the *Observation* resource as a semantically rich, queryable index that references externally stored raw signals. The resulting profiles provide a standardized method for cataloging, querying, and analyzing continuous monitoring data within the FHIR ecosystem, providing a standards-compliant alternative to proprietary formats and fragmented data silos.

The core deliverable, the TIDE *Observation* profile, achieves the primary objective of decoupling metadata from bulk signal storage through the *Metadata-Proxy* pattern, enforced via a mandatory *RawDataEndpoint* extension and complemented by an optional *PreviewEndpoint* for lightweight visualization. The 16 component *slices*, organized across acquisition parameters, statistical summaries, and advanced signal-processing metrics, enable FHIR-level signal quality filtering without raw data retrieval—a capability not available in existing FHIR-native approaches. The explicit semantic distinction between technical artifacts (*anomalyCount*) and physiological events (*eventCount*), combined with metrics such as *samplingJitter*, *dominantFrequency*, and *signalEntropy*, positions TIDE as a lightweight integration layer that can be embedded within preprocessing or postprocessing stages of existing data pipelines, enabling quality-aware access and standardized exchange of continuous monitoring data in clinical research or AI-driven workflows.

### Comparison With Prior Work

Existing FHIR-based implementations have explored multiple strategies for representing medical monitoring data [1,11,14,35]. However, no single approach fully supports maintaining the continuous nature of the data with efficient storage, semantic accessibility, and querying within the FHIR framework.

A conventional strategy represents high-frequency measurements as individual FHIR *Observation* resources with discrete timestamps [36]. While this approach preserves native FHIR semantics and enables straightforward querying via standard APIs, it scales poorly for dense monitoring data because it generates a large number of resources, placing considerable strain on conventional FHIR server infrastructure. In parallel, routine clinical practice continues to rely heavily on document-centric or proprietary formats for continuous monitoring data, particularly PDF reports. This continued use of document-based exchange, even where structured interoperability standards are available, reflects pragmatic constraints and heterogeneous clinical IT landscapes [37].

To accommodate such practices, document-centric strategies have been proposed in which high-frequency medical monitoring data are stored as external artifacts and linked via FHIR *DocumentReference* or Binary resources, with the FHIR layer providing contextual metadata such as patient association and document type [38,39]. This design reduces server burden and supports scalable integration with file systems or object storage backends. However, because the internal payload structure is not interpreted at the FHIR level, the data remain semantically opaque, preventing fine-grained querying and automated filtering. Consequently, downstream systems must retrieve and parse the complete dataset before determining its suitability for clinical or research use. Although effective for archival exchange and legacy system integration, this pattern treats high-frequency medical monitoring data as static documents rather than continuous, queryable signals, limiting its applicability for longitudinal monitoring and secondary use [37]. The proposed TIDE approach addresses these limitations by avoiding treating high-frequency medical monitoring data as opaque binary payloads. By linking acquisition parameters and derived analytics directly to the *Observation* resource, TIDE preserves the semantic accessibility and queryability of high-frequency medical monitoring data, supporting secondary use. As a result, a key architectural strength of TIDE profiling is its storage-agnostic design, which provides a high degree of independence from the underlying persistence layer. By abstracting data access through the *Endpoint* resource, the framework ensures that the choice of backend technology (eg, cloud-native object storage such as Amazon S3 or MinIO) and specialized, high-performance databases (eg, TimescaleDB, QuestDB) remains entirely transparent to the clinical interoperability layer. The HL7 FHIR *SampledData* data type has been proposed as a native representation of high-frequency medical monitoring data within the *Observation* resource. *SampledData* embeds medical monitoring data as a character string accompanied by interpretive metadata, such as signal origin, sampling period, scaling factor, and optional value bounds, enabling semantic self-description and unit-consistent interpretation of physiological signals [14,27]. However, the nested encoded JSON format requires complete deserialization before analysis, which prevents efficient database-level scans and vectorized operations on the signal content [1]. As a result, this architectural constraint becomes problematic in high-velocity streaming and large-scale longitudinal analytics scenarios and motivates transformation into columnar representations better suited to time series processing [1]. In contrast, TIDE decouples storage concerns, designed to remain lightweight and readily searchable, as well as acting as a catalog entry. The heavy lifting of storage is offloaded to specialized high-frequency monitoring databases or object storage systems optimized for high-throughput streaming. This separation of concerns is intended to support scalability and aligns with modern cloud-native architectures.

Beyond the core FHIR data types, several specialized approaches have sought to bridge signal-processing–oriented data models with FHIR-based interoperability. For example, stream-enabled annotated ECG represents a targeted evolution of the HL7 Annotated ECG standard by using a “timestamp difference” encoding, which records the intervals between successive samples rather than absolute timestamps [11]. This approach substantially reduces data volume and supports long-term monitoring. Similarly, hybrid formats, such as SCP-ECG and PDF-ECG, combine FHIR-based metadata with established, highly compressed formats [40]. Although technically efficient, these approaches are typically domain-specific and operate largely outside the generic FHIR profiling ecosystem, limiting uniform longitudinal querying across different medical modalities [41]. Furthermore, while the EDF remains the global standard for clinical neurophysiology and sleep research, it is typically handled as an isolated binary artifact within current clinical IT landscapes [10]. TIDE bridges this gap by extracting and indexing technical signal properties directly into the FHIR *Observation* resource, allowing clinical systems to evaluate signal suitability via standard FHIR search parameters without the computational overhead of first downloading and parsing the entire high-fidelity file, thereby reducing fragmentation across signal domains and supporting cross-disciplinary interoperability.

More details on the practical comparison between TIDE and existing approaches are provided in Multimedia Appendix 3. These architectural comparisons are conceptual; the corresponding quantitative comparison and its limitations are also reported in Multimedia Appendix 3.

### Design Rationale

The TIDE framework required several nontrivial architectural decisions for which FHIR offers multiple technically valid alternatives. The following sections document the rationale for 3 key choices: using *component slicing* rather than dedicated *Observation* resources, using lightweight extensions rather than *DocumentReference* for external data linkage, and designing adaptable rather than prescriptive terminology artifacts.

In the flat TIDE *Observation* model, we preferred a one-slice-per-metric approach using 16 component slices for technical metadata, statistical summaries, and data quality metrics rather than dedicated *Observation* resources. The advantages of component slicing include avoiding data structure redundancy and the potential for improved internal consistency and transactional integrity, as all related elements are maintained within a single resource instance. They can be retrieved and consumed together as a single result set via the Python client library (such as FHIR PYrate [42]), which could be used to retrieve data from the clinical FHIR server via the FHIR RESTful API. Component-level metrics in the flat model remain searchable via the standard composite search parameter *component-code-value-quantity* [43], which ties the matched code and value to the same component. However, because the flat *Observation* identifies the channel only through *component.code.text*, which is not covered by this search parameter, such matches cannot be attributed to individual electrodes. This gap is addressed by a complementary hierarchical model, in which per-channel child *Observations* linked to a parent via *hasMember* are each independently queryable. This approach enables electrode-level metric filtering where required; for example, to retrieve all recordings in which a specific channel’s *signalStabilityIndex* falls below a predefined threshold. The two patterns are therefore complementary: the flat model prioritizes transactional efficiency and single-query retrieval for high-channel-count recordings, while the hierarchical model prioritizes per-channel discoverability at the cost of additional resource instances.

Another design decision in this FHIR package concerns the use of extensions with the TIDE *Endpoint* profile rather than the *DocumentReference* resource. Although *DocumentReference* supports externally stored documents, raw data are often maintained in specialized storage backends whose performance benefits may be diminished by binary encapsulation. Our lightweight extension approach acts as a semantic pointer that keeps the *Observation* resource lean and is intended to reduce operational overhead when the raw payload is only selectively required. A lightweight extension referencing the dedicated *TIDEEndpoint* profile was consequently adopted as the more proportionate solution, maintaining semantic precision without the administrative overhead of document management.

In addition, the terminology definitions in this package were derived from our institutional requirements and use cases. Associated artifacts were intentionally designed as extensible rather than prescriptive, reflecting 2 considerations: first, no established consensus exists for technical signal-processing metadata in international vocabularies, necessitating a pragmatic starting point; second, FHIR’s extensibility model explicitly supports this pattern, allowing *CodeSystems* and *ValueSets* to be profiled and extended without breaking conformance. This approach prioritizes practical deployability over premature standardization, with the expectation that community feedback will inform future consolidation.

### Clinical Implications

TIDE supports longitudinal patient monitoring by representing each recording segment as a self-contained, time-bounded *Observation* resource with explicit start and end timestamps via the *effectivePeriod* element. The segmentation strategy, in which recording intervals within a session are assigned sequential *preliminary* status until session completion (flat model), ensures that longitudinal datasets are both temporally precise and traceable. This enables systematic comparison of derived analytical metrics across care transitions, readmissions, or multisite studies, for example, tracking signal entropy as an indicator of neurophysiological complexity or dominant frequency shifts as markers of evolving clinical states over extended monitoring periods.

This profile is also well aligned with AI-driven clinical and research workflows, as the proposed structure enables efficient metadata management in lakehouse and data lake architectures by maintaining a lean FHIR layer while raw signals are stored in purpose-built backends [44]. Although global adoption of FHIR has increased substantially, integrating high-frequency data for AI training remains challenging due to the complexity of parsing nested resource payloads [1,45]. TIDE addresses this by exposing a lightweight, metadata-based index via queryable FHIR elements. This allows researchers to select cohorts based on signal characteristics before retrieving bulk data, thereby supporting scalable and standardized data pipelines for longitudinal digital health studies. Furthermore, the storage-agnostic *Endpoint* architecture enables multicenter deployments in which participating institutions maintain their own signal repositories while exposing a uniform FHIR metadata interface.

The queryable analytical metrics provided by the TIDE profile could further enable automated clinical triage workflows. Threshold-based algorithms can systematically identify recordings that meet predefined criteria. For example, algorithms could flag all segments with *anomalyCount* exceeding a defined threshold for targeted clinical review or filter cohorts by *dataCoverage* to exclude low-quality recordings from downstream analysis. This enables scalable, automated prioritization of clinically relevant data in high-volume monitoring environments.

### Limitations and Future Work

The empirical evaluation reflects a PoC scope and included data from 2 publicly available EEG datasets described in Multimedia Appendix 2, comprising 4 subjects and 5 EDF recordings. Although these datasets were deliberately selected to capture differences in acquisition scales, such as sampling frequency or channel count, and channel-naming conventions, the sample size remains small and limited to EEG.

Additionally, samplingJitter reflects the nominal timing precision of the source file format rather than true acquisition-hardware jitter; for formats such as EDF, which do not retain per-sample timestamps, this metric is structurally bounded and will read as zero regardless of true hardware timing stability. Broader validation across additional modalities, larger cohorts, and multisite data sources will be necessary to further establish the generalizability and robustness of TIDE across clinical monitoring environments.

The TIDE framework provides structured metadata and standardized external data references but does not prescribe the format for the underlying raw data payload, which may vary across EDF, a Parquet file, or a proprietary binary format. Although TIDE metadata facilitates interpretation and parsing, payload-level interoperability still depends on agreement between data producers and consumers. Moreover, the current *TIDEEndpoint* profile and the PoC deployment specify payload location, connection protocol, and payload format but do not address authentication or authorization. Versioning is limited to changes through Endpoint.meta.versionId and does not track versions of the referenced raw payload (see Multimedia Appendix 2) . Future work should address standardized payload representation and production-oriented security and provenance mechanisms, such as authentication or authorization extensions and payload checksums.

Although the *TIDECodeSystem*, the *TIDESupplementalCodes CodeSystem*, the *TIDEValueSet,* and the additional *TIDEChannelCodeSystem* as well as *TIDEConnectionTypeCodes* were defined to support semantic consistency within the profile, these terminological artifacts do not exhaustively cover all potentially relevant clinical and device-specific scenarios. For instance, emerging wearable modalities and highly specialized neuromonitoring signals may require additional codes not yet present in the current definitions, and formal alignment with device-level interoperability standards such as IEEE 11073 (medical device communication standards) was beyond the scope of the study. Future work will therefore concentrate on systematically extending and harmonizing the terminology framework to accommodate emerging wearable modalities and highly specialized neuromonitoring signals, aligning it with IEEE 11073 terminology, and integrating it with *SMART on FHIR* applications [46] for real-time dashboarding.

Beyond structural and terminological refinements, future work should also address advanced retrieval strategies. More granular *SearchParameter* definitions and standardized query patterns, potentially using Clinical Quality Language, could enhance data retrieval consistency for specific TIDE components across implementations and strengthen operational interoperability. The current PoC treats each preexisting recording file as a single segment and does not address continuous-recording lifecycle management, including fixed-window segmentation splitting algorithms, overlapping and duplicated segment boundary detection, or late-arriving data recognition. These capabilities will be important for future production deployment ingesting truly continuous, real-time streams.

### Conclusions

The TIDE package provides a reusable, standards-compliant framework for integrating complex high-frequency monitoring data into the FHIR ecosystem. By decoupling metadata from raw data and enforcing semantic precision in signal quality metrics, TIDE bridges the gap between advanced monitoring technologies and interoperable health care data exchange. This approach lays the foundation for longitudinal analysis and the seamless integration of automated, algorithmic analysis results into routine clinical care.

## Supplementary material

10.2196/94282 Multimedia Appendix 1 Channel reference.

10.2196/94282 Multimedia Appendix 2 Real-world data implementation.

10.2196/94282 Multimedia Appendix 3 Practical comparison.

## Abbreviations

ECG: electrocardiography
EDF: European Data Format
EEG: electroencephalography
FHIR: Fast Healthcare Interoperability Resources
HL7: Health Level 7
LOINC: Logical Observation Identifiers Names and Codes
PoC: proof-of-concept
R4: Release 4
RESTful API: Representational State Transfer Application Programming Interface
SNOMED CT: Systematized Nomenclature of Medicine Clinical Terms
TIDE: Time Series Integration and Data in Endpoints
