# Supplementary material for: Exchange of High-Frequency Medical Monitoring Data Using Fast Healthcare Interoperability Resources for Interoperable Integration Into Clinical Practice and Research: Methodological Development Study
Source: JMIR Med Inform. 2026 Nov 2;14:e94282. doi: 10.2196/94282 (PMC13727023; doi:10.2196/94282)
Supplement: Supplementary file 1 [file medinform-v14-e94282-s001.docx]

Channel reference

# Background

In multichannel physiological recordings such as EEG, signal quality metrics are inherently channel-specific. Metrics such as dominant frequency or entropy may vary substantially across electrode locations, reflecting both physiological gradients and electrode contact quality. Accordingly, a key design requirement for TIDE was to support explicit binding of analytical metrics to the specific signal channel from which they were derived, enabling channel-level querying and filtering without requiring retrieval of the complete recording.

# Parent-Child Observation Model for Channel-Level Metric Binding

TIDE addresses this requirement through a hierarchical parent-child Observation structure. A single parent TIDE Observation resource captures global recording parameters via its component slices and references individual per-channel child Observation resources through the standard FHIR R4 hasMember element. Each child Observation encodes the analytical metrics, as shown in Table M2.2, for a specific channel and identifies that channel via the structured bodySite element, extensibly bound to the *TIDEChannelValueSet* (which includes all codes from the *TIDEChannelCodeSystem*; see Table M2.1), with the original source channel label preserved in bodySite.text. Its code element carries the fixed canonical text “EEG study” (LOINC 11523-8), unrelated to channel identity (see also: Multimedia Appendix 2).

**Table M2.1.** TIDEChannelCodeSystem: codes for channel/electrode identity.

| **Category** | **Codes** | **Description** |
| --- | --- | --- |
| Standard International 10-20 System positions (21) | Fp1, Fp2, F7, F3, Fz, F4, F8, T3, C3, Cz, C4, T4, T5, P3, Pz, P4, T6, O1, O2, A1, A2 | Standard EEG electrode positions per the International 10-20 System (Jasper, 1958); legacy labels T3/T4/T5/T6 correspond to modern equivalents T7/T8/P7/P8. |
| Extended nasopharyngeal positions (2) | Pg1, Pg2 | Left/right nasopharyngeal electrode positions from the extended 10-20 nomenclature, used in some clinical epilepsy/sleep montages. |
| Fallback (1) | non-standard-channel | Used when a channel label does not map to a recognized electrode position (e.g., generic or anonymized labels such as EEG001); the original label is preserved in bodySite.text. |

**Table M2.2.** Summary of Channel-level metrics represented in child Observation.

| **Component (TIDECodeSystem code)** | **Description** | **Value type** | **Unit** |
| --- | --- | --- | --- |
| signalMean | Arithmetic mean of channel amplitude | Quantity | µV |
| signalMin | Minimum channel amplitude | Quantity | µV |
| signalMax | Maximum channel amplitude | Quantity | µV |
| signalStdDev | Standard deviation of channel amplitude | Quantity | µV |
| signalStabilityIndex^a^ | Amplitude stability (coefficient-of-variation-style ratio) | Quantity | dB |
| dominantFrequency | Frequency with the highest power spectral density (Welch method) | Quantity | Hz |
| trendSlope | Slope of signal baseline drift | Quantity | µV/s |
| signalEntropy | Shannon entropy of amplitude distribution | Quantity | bit |
| samplingJitter | Standard deviation of sampling intervals | Quantity | ms |
| anomalyCount | Number of detected technical artifacts | Integer | — |

^a^ signalStabilityIndex is calculated with 20·log10 ( | mean | / SD ) of the raw amplitudes of a channel.

We did not involve signal-to-noise (SNR) measures in this paper, because a spectral SNR would require a modality-specific definition of the expected physiological frequency band, which would not generalize across the heterogeneous signal types of TIDE without introducing modality-specific parameters into an otherwise generically developed profile. Note that for zero-mean or near-zero-mean alternating-current (AC)-coupled signals (e.g., high-pass-filtered or baseline-corrected EEG/ECG), the signalStabilityIndex is not monotonic with perceived signal quality. A stable, well-referenced baseline (against zero) yields a low, even negative value, whereas a larger, constant direct-current (DC) offset can yield higher values. Higher variance in the physiological signals also lowers the ratio. Therefore, we use the metric signalStabilityIndex to measure baseline/DC stability relative to signal dispersion, but not as an overall indicator of the quality of neuronal or cardiac signals.

While each child Observation encodes channel-specific 10 component slices drawn from the TIDECodeSystem, the parent Observation retains six global component slices (samplingFrequency, sampleCount, channelCount, dataCoverage, dataGaps, eventCount) that apply to the recording as a whole.

As shown in Table M2.3, a single parent Observation is created for each recording, while a child Observation (*TIDEObservationChild)* is generated for each channel.

**Table M2.3.** Observation structure and component allocation per recording.

| **Level** | **Resource type** | **Content** | **Count (per recording)** |
| --- | --- | --- | --- |
| Parent | TIDEObservation (profile) | 6 global component slices | 1 |
| Child | TIDEObservationChild (profile) | 10 per-channel component slices | N (one per EEG channel) |

The parent and child resources share the same subject reference (Patient), enabling a single subject-scoped FHIR query to retrieve the complete parent-child set, making each channel's metrics independently accessible via FHIR search, using the standard component-code-value-quantity parameter for metrics and a custom bodysite *SearchParameter* for electrode filtering (see Multimedia Appendix 2).

# Implementation Example

The hierarchical model is applied to three subjects from the OpenNeuro dataset ds007823, as described in Multimedia Appendix 2. Because the number of EEG recording channels varied across subjects, the resulting FHIR resource structures differed slightly. Table M2.4 summarizes the EEG channels represented within the generated FHIR resources for each subject.

**Table M2.4.** Overview of EEG channels.

| **Subject** | **EEG channels (N)** | **Channel names (source labels)** |
| --- | --- | --- |
| sub-CUCOV003 | 21 | Fp1, Fp2, F7, F3, FZ, F4, F8, T3, C3, CZ, C4, T4, T5, P3, PZ, P4, T6, O1, O2, Pg1, Pg2 |
| sub-CUCOV008 | 21 | Fp1, Fp2, F7, F3, FZ, F4, F8, T3, C3, CZ, C4, T4, T5, P3, PZ, P4, T6, O1, O2, Pg1, Pg2 |
| sub-CUCOV020 | 22 | Fp1, Fp2, F7, F3, FZ, F4, F8, T3, C3, CZ, C4, T4, T5, P3, PZ, P4, T6, O1, O2, Pg1, Pg2, 25+ |

Note: sub-CUCOV020 includes a non-standard channel label ("25+") as present in the source EDF file; this label is preserved in bodySite.text, while bodySite.coding is mapped to the fallback code non-standard-channel in the TIDEChannelCodeSystem, since “25+” does not correspond to a recognized International 10-20 System position.

The following excerpt illustrates a child Observation for the Fp1 electrode of sub-CUCOV003, as stored in the Blaze FHIR server. Non-essential fields (id, meta) are omitted for brevity.

```
{
 "resourceType": "Observation",
 "status": "final",
 "code": {
   "coding": [
     {
       "system": "http://loinc.org",
       "code": "11523-8",
       "display": "EEG study"
     }
   ],
   "text": "EEG study"
 },
 "subject": {
   "reference": "Patient/DHV3WIJIPDJIH64I"
 },
 "effectivePeriod": {
   "start": "2026-01-01T12:41:55Z",
   "end": "2026-01-01T12:55:24.995Z"
 },
 "bodySite": {
   "coding": [
     {
       "system": "http://example.org/tide/CodeSystem/tide-channel-code-system",
       "code": "Fp1",
       "display": "Fp1"
     }
   ],
   "text": "Fp1"
 },
 "method": {
   "text": "TIDE analytical metrics computed via compute_slices() (numpy/scipy Welch PSD, MNE-based EEG preprocessing)."
 },
 "device": {
   "reference": "Device/DIGYKNL24KC3GARU"
 },
 "derivedFrom": [
   {
     "reference": "Observation/e00b6a90-a1b9-4320-b79c-37bdab122b90"
   }
 ],
 "component": [
   {
     "code": {
       "coding": [
         {
           "system": "http://example.org/tide/CodeSystem/tide-code-system",
           "code": "signalMean",
           "display": "Signal mean"
         }
       ]
     },
     "valueQuantity": {
       "value": -36.35196,
       "unit": "uV",
       "system": "http://unitsofmeasure.org",
       "code": "uV"
     }
   },
   {
     "code": {
       "coding": [
         {
           "system": "http://example.org/tide/CodeSystem/tide-code-system",
           "code": "signalMin",
           "display": "Signal Minimum"
         }
       ]
     },
     "valueQuantity": {
       "value": -183.333327,
       "unit": "uV",
       "system": "http://unitsofmeasure.org",
       "code": "uV"
     }
   },
   {
     "code": {
       "coding": [
         {
           "system": "http://example.org/tide/CodeSystem/tide-code-system",
           "code": "signalMax",
           "display": "Signal Maximum"
         }
       ]
     },
     "valueQuantity": {
       "value": 61.580552,
       "unit": "uV",
       "system": "http://unitsofmeasure.org",
       "code": "uV"
     }
   },
   {
     "code": {
       "coding": [
         {
           "system": "http://example.org/tide/CodeSystem/tide-code-system",
           "code": "signalStdDev",
           "display": "Signal Standard Deviation"
         }
       ]
     },
     "valueQuantity": {
       "value": 13.922502,
       "unit": "uV",
       "system": "http://unitsofmeasure.org",
       "code": "uV"
     }
   },
   {
     "code": {
       "coding": [
         {
           "system": "http://example.org/tide/CodeSystem/tide-code-system",
           "code": "signalStabilityIndex",
           "display": "Signal Stability Index"
         }
       ]
     },
     "valueQuantity": {
       "value": 8.3362,
       "unit": "dB",
       "system": "http://unitsofmeasure.org",
       "code": "dB"
     }
   },
   {
     "code": {
       "coding": [
         {
           "system": "http://example.org/tide/CodeSystem/tide-code-system",
           "code": "dominantFrequency",
           "display": "Dominant Frequency"
         }
       ]
     },
     "valueQuantity": {
       "value": 0.293,
       "unit": "Hz",
       "system": "http://unitsofmeasure.org",
       "code": "Hz"
     }
   },
   {
     "code": {
       "coding": [
         {
           "system": "http://example.org/tide/CodeSystem/tide-code-system",
           "code": "trendSlope",
           "display": "Signal Trend Slope"
         }
       ]
     },
     "valueQuantity": {
       "value": -0.000296,
       "unit": "uV/s",
       "system": "http://unitsofmeasure.org",
       "code": "uV/s"
     }
   },
   {
     "code": {
       "coding": [
         {
           "system": "http://example.org/tide/CodeSystem/tide-code-system",
           "code": "signalEntropy",
           "display": "Signal Entropy"
         }
       ]
     },
     "valueQuantity": {
       "value": 4.355428,
       "unit": "bit",
       "system": "http://unitsofmeasure.org",
       "code": "bit"
     }
   },
   {
     "code": {
       "coding": [
         {
           "system": "http://example.org/tide/CodeSystem/tide-code-system",
           "code": "samplingJitter",
           "display": "Sampling Jitter"
         }
       ]
     },
     "valueQuantity": {
       "value": 0.0,
       "unit": "ms",
       "system": "http://unitsofmeasure.org",
       "code": "ms"
     }
   },
   {
     "code": {
       "coding": [
         {
           "system": "http://example.org/tide/CodeSystem/tide-code-system",
           "code": "anomalyCount",
           "display": "Technical Anomaly Count"
         }
       ]
     },
     "valueInteger": 2205
   }
 ]
}
```

**Figure M2.1.** Representative example (excerpt) of a child Observation for the Fp1 electrode. The CodeSystem URLs (http://example.org/tide/CodeSystem/tide-code-system, http://example.org/tide/CodeSystem/tide-channel-code-system) are placeholders used during development and will be updated to the final published IG canonical URLs upon acceptance.
