# Supplementary material for: Exchange of High-Frequency Medical Monitoring Data Using Fast Healthcare Interoperability Resources for Interoperable Integration Into Clinical Practice and Research: Methodological Development Study
Source: JMIR Med Inform. 2026 Nov 2;14:e94282. doi: 10.2196/94282 (PMC13727023; doi:10.2196/94282)
Supplement: Supplementary file 2 [file medinform-v14-e94282-s002.docx]

Real-world data implementation

# Overview

To assess the feasibility of the TIDE framework in a realistic scenario, a proof-of-concept implementation was conducted using two publicly available EEG datasets from the OpenNeuro repository [1,2]. The two datasets were selected to demonstrate the framework's complementary strengths: high-frequency scalability with the flat single-Observation model and clinically relevant per-channel electrode granularity using the hierarchical parent/child model. Both datasets targeted the same FHIR server instance.

# Demonstration Datasets

Dataset ds007808 is an EEG-Speech Brain Decoding Dataset containing high-density EEG recordings collected using multiple acquisition systems with varying channel counts [1]. Each subject session in this dataset comprised multiple EDF files, and for this implementation, a single subject (sub-03) was used. The subject comprised two EDF files from a single session, corresponding to a speech perception paradigm (task-speechopen). Dataset ds007823 [2] contributed three subjects to this implementation (sub-CUCOV003, sub-CUCOV008, sub-CUCOV020). Each subject provided one EDF file at 200 Hz with 21–22 EEG channels using standard 10-20 system electrode names.

# Data Preprocessing and FHIR Conversion

All EDF files were read using MNE-Python [[3]](https://www.zotero.org/google-docs/?wMnz8k). For data from ds007808, signal data were segmented into the full recording window per run. For each run, the following 16 TIDE component slices were computed: global acquisition parameters (samplingFrequency, sampleCount, channelCount), statistical summaries (signalMean, signalMin, signalMax, signalStdDev), and data quality metrics (dataCoverage, dataGaps, signalStabilityIndex, samplingJitter, dominantFrequency, trendSlope, signalEntropy, anomalyCount, eventCount). Statistical summaries and quality metrics were computed for each EEG channel using NumPy [[4]](https://www.zotero.org/google-docs/?DtpxYZ) and SciPy [[5]](https://www.zotero.org/google-docs/?B3hiBk), while the dominant frequency was derived from the power spectral density. Global acquisition parameters were computed across the full recording. All per-channel components and global slices were encoded as component entries in a single flat TIDE Observation resource that conforms to the TIDE Observation profile. To demonstrate the status lifecycle under the incremental-representation assumption (as described in the manuscript), the status of run-01 was set to preliminary to represent an ongoing monitoring session; run-02 was set to final. Because the EDF header was anonymized, the start of each effectivePeriod was set to the BIDS session date (00:00 UTC) and the end was derived from the recording duration; the effectivePeriod therefore reflects the recording duration, not the actual recording time.

For data from ds007823, the hierarchical model was applied: a parent TIDE Observation resource was created per subject, encoding six global component slices (samplingFrequency, sampleCount, channelCount, dataCoverage, dataGaps, eventCount). For each EEG channel, a separate child Observation conforming to the *TIDEObservationChild* profile was created, encoding 10 per-channel component slices (signalMean, signalMin, signalMax, signalStdDev, signalStabilityIndex, samplingJitter, dominantFrequency, trendSlope, signalEntropy, anomalyCount). Each child’s code element carries the fixed canonical text “EEG study” (LOINC 11523-8) in both code.coding.display and code.text. Channel/electrode identity is instead encoded separately in the structured bodySite element, coded from the TIDEChannelCodeSystem (e.g., Fp1) via the extensibly bound TIDEChannelValueSet (see Multimedia Appendix 1), with the original source channel label preserved in bodySite.text. The parent referenced all children via the standard FHIR hasMember element. Children carried a subject reference to the same Patient resource as the parent, enabling standard subject-scoped FHIR queries to retrieve the complete parent-child set.

Each Observation in the flat model, or parent Observation in the hierarchical model, references a raw-data Endpoint resource via the tide-rawdata-endpoint Extension, conforming to the TIDEEndpoint profile. For both datasets used in this proof-of-concept, Endpoint.address resolves to the public, unauthenticated OpenNeuro repository URL for the corresponding EDF file, enabling a consuming system to determine the data location, connection protocol, and payload format directly from the Endpoint resource.

The current implementation is limited to a publicly accessible, unauthenticated data source and therefore does not address access control required for non-public clinical data. In production settings, the address element would naturally resolve to an access-controlled institutional storage backend, such as a PACS or object storage system. Table M1.1 summarizes the operational endpoint aspects represented by the corresponding FHIR elements and distinguishes those demonstrated in the current implementation from those requiring further consideration for production deployment.

**Table M1.1.** Operational endpoint elements and proof-of-concept implementation status

| **Aspect** | **FHIR element** | **Status** |
| --- | --- | --- |
| Payload location | Endpoint.address | Demonstrated (public OpenNeuro URL, live example) |
| Access method/protocol | Endpoint.connectionType | Demonstrated (coded direct-https, see TIDEConnectionTypeCodes) |
| File format | Endpoint.payloadMimeType, payloadType | Demonstrated (application/edf, eegWaveform) |
| Authentication/authorization | (no element in profile) | Not addressed; required for access-controlled deployments (e.g. institutional PACS/object storage), not implemented here |
| Payload versioning | Endpoint.meta.versionId | Partial; tracks changes to the Endpoint resource itself, not the referenced raw file |
| Data integrity | (no element in profile) | Not addressed; no checksum/hash of the referenced payload |

# Results and Evaluation

## FHIR Resources Generation

All FHIR R4 resources from both data sources were stored in a locally deployed instance of the Blaze FHIR server and accessed via its RESTful API. Table M1.2 summarizes the number of resources created per resource type for each data source, along with the combined total across both datasets.

**Table M1.2.** FHIR Resource Counts.

| Resource type | Data sources | Count | Notes |
| --- | --- | --- | --- |
| Patient | ds007808 | 1 | sub-03;  identified via BIDS subject label |
|  | ds007823 | 3 | One per subject |
|  | Total | 4 |  |
| Device | ds007808 | 2 | One per run;  manufacturer: g.tec medical engineering GmbH;  model: g.Pangolin |
|  | ds007823 | 3 | One per subject |
|  | Total | 5 |  |
| Endpoint | ds007808 | 2 | One per run;  address: public OpenNeuro repository URL |
|  | ds007823 | 3 | One per subject;  address: public OpenNeuro repository URL |
|  | Total | 5 |  |
| Observation (TIDE) | ds007808 | 2 | One per run;  1,286 components each  (6 global + 128 channels × 10 per-channel slices) |
|  | ds007823 | 3 | Observation Parent  One per subject;  6 global components each |
|  |  | 64 | Observation Child  21, 21, 22 channels respectively;  10 per-channel components each |
|  | Total | 69 |  |
| Total | ds007808 | 7 |  |
|  | ds007823 | 76 |  |
|  | Total | 83 |  |

## FHIR Mapping Evaluation

To assess the correctness and completeness of the TIDE resource population, precision and recall were computed by comparing TIDE-extracted values with ground-truth fields in the BIDS sidecar JSON files. Precision was defined as the fraction of TIDE-populated fields that were verified to be correct, and recall was defined as the fraction of verified sidecar fields that TIDE captured. Numeric values were compared with a 1% relative tolerance; string values were compared case-insensitively. The two datasets were evaluated independently because the available sidecar fields differ.

For ds007808, as shown in Table M1.3, five sidecar fields were available for verification: SamplingFrequency, EEGChannelCount, Manufacturer, ManufacturersModelName, and PowerLineFrequency. TIDE populated the first four via the Observation component slices (samplingFrequency, channelCount) and the referenced Device resource (manufacturer, model name). PowerLineFrequency is a hardware configuration value, not a signal quality metric, and is therefore intentionally absent from the TIDE component slice definitions.

**Table M1.3**. Precision and Recall of FHIR Mapping for sub-03 from ds007808.

| Run | GT fields available | TIDE populated | Correct | Precision | Recall |
| --- | --- | --- | --- | --- | --- |
| run-01 | 5 | 4 | 4 | 1.00 | 0.80 |
| run-02 | 5 | 4 | 4 | 1.00 | 0.80 |
| Mean |  |  |  | 1.00 | 0.80 |

For ds007823, as shown in Table M1.4, three sidecar fields were available: SamplingFrequency, EEGChannelCount, and PowerLineFrequency. No Manufacturer or ManufacturersModelName was present in the sidecar. TIDE populated the first two via the parent Observation's global component slices. PowerLineFrequency was again absent by design.

**Table M1.4**. Precision and Recall of FHIR Mapping for data from ds007823.

| Subject | GT fields available | TIDE populated | Correct | Precision | Recall | Child observations |
| --- | --- | --- | --- | --- | --- | --- |
| sub-CUCOV003 | 3 | 2 | 2 | 1.00 | 0.67 | 21 |
| sub-CUCOV008 | 3 | 2 | 2 | 1.00 | 0.67 | 21 |
| sub-CUCOV020 | 3 | 2 | 2 | 1.00 | 0.67 | 22 |
| Mean |  |  |  | 1.00 | 0.67 |  |

In conclusion, precision reached 1.00 across all recordings and both datasets, indicating that every value TIDE extracted was verifiably consistent with the BIDS ground truth. Recall that below 1.00 in both cases reflect the deliberate exclusion of PowerLineFrequency from the TIDE component slice definitions, as this parameter describes hardware configuration rather than signal quality.

## Metric Ground-Truth Validation

To assess the correctness of the 16 analytical metrics independently of the BIDS sidecar comparison, we validated the core signal-analysis function, compute_slices, against synthetically generated signals with known, analytically derived properties. Real EEG recordings have no independent ground truth for these computed metrics, so synthetic test signals are the standard validation approach. All 17 validation checks passed, covering 15 of the 16 metrics; *eventCount* was not included in the synthetic validation.

**Table M1.5** Validation results.

| metric | case | expected | observed | status |
| --- | --- | --- | --- | --- |
| signalMean | linspace(-500,1500) | 500.0 | 500.0 | PASS |
| signalMin | linspace(-500,1500) | -500.0 | -500.0 | PASS |
| signalMax | linspace(-500,1500) | 1500.0 | 1500.0 | PASS |
| signalStdDev | linspace(-500,1500) | 577.4657 | 577.4657 | PASS |
| signalStabilityIndex | linspace(-500,1500) | -1.25 | -1.25 | PASS |
| samplingFrequency | linspace(-500,1500) | 250.0 | 250.0 | PASS |
| sampleCount | linspace(-500,1500) | 5000 | 5000 | PASS |
| channelCount | single-channel record | 1 | 1 | PASS |
| dataCoverage | no NaNs | 100.0 | 100.0 | PASS |
| dataGaps | no annotations | 0 | 0 | PASS |
| samplingJitter | uniform sfreq (mne RawArray) | 0.0 | 0.0 | PASS |
| dominantFrequency^a^ | 10 Hz sine + N(0,2.0) noise (Gaussian) | 10.0 | 10.0098 | PASS |
| anomalyCount | 7 injected 500µV outliers in U(-5,5) baseline | 7 | 7 | PASS |
| dataCoverage | 50/1000 samples NaN | 95.0 | 95.0 | PASS |
| dataGaps | 3 injected boundary/edge annotations | 3 | 3 | PASS |
| trendSlope | pure linear ramp, 3.0 µV/s | 3.0 | 3.0 | PASS |
| signalEntropy^b^ | sine (10Hz) vs Gaussian noise, same amplitude | entropy (noise) >(sine) | sine=4.5191, noise=5.8047 | PASS |

^a^ a 0.0098 Hz difference is counted as a match, because it is within the Welch PSD frequency resolution (0.061 Hz)
^b^ no exact target or numeric match; PASS solely reflects the correct directional relationship, where

noise entropy > sine entropy

In addition, Table M1.6 reports the calculation methods for the six recording-level metrics; per channel-metric calculations are documented in Multimedia Appendix 1, Table M2.2.

**Table M1.6** Calculation methods of the six recording-level metrics.

| **Metric** | **Calculation Method** |
| --- | --- |
| samplingFrequency | EDF sampling rate as reported by MNE [Hz] |
| sampleCount | Number of time points in the recording |
| channelCount | Number of the EEG channels selected for analysis |
| dataCoverage | Percentage of non-NaN samples across all selected channels and time points |
| dataGaps | Count of MNE annotations whose descriptions contain “boundary” or “edge”, marking data discontinuity |
| eventCount | Direct row count of the BIDS _events.tsv file, no calculation |

## Profile Conformance Validation

All generated FHIR example instances were validated against their declared StructureDefinition profiles using the official HL7 FHIR IG Publisher. Channel identity is stored in component.code.text (flat model) or the structured bodySite element (hierarchical model). The IG Publisher reported zero validation errors for all example instances.

Example FHIR Queries

The following queries were executed against the Blaze FHIR server to demonstrate standard FHIR search capabilities over the TIDE resources. Patient IDs were resolved at runtime via Patient?identifier=<subject-label> before subject-scoped queries, because Blaze does not execute chained identifier searches (subject.identifier=) without additional index configuration.

**Table M1.7.** Overview of FHIR Query Examples.

| Query | FHIR URL | Results |
| --- | --- | --- |
| All Observations for sub-03 (flat model) | Observation?subject=Patient/<id>&_count=50 | 2 |
| Preliminary-status Observation | Observation?status=preliminary&_count=50 | 1 |
| All Observations for sub-CUCOV003 (parent + 21 children) | Observation?subject=Patient/<id>&_count=50 | 22 |
| Cross-dataset: all Observations from 1985-01-01 onwards | Observation?date=ge1985-01-01&_count=100 | 69 |
| signalStabilityIndex < 0 dB (composite search parameter) | Observation?component-code-value-quantity=http://example.org/tide/CodeSystem/tide-code-system\|signalStabilityIndex$lt0&_count=50 | 3 |
| Data coverage ≥ 100% | Observation?component-code-value-quantity=http://example.org/tide/CodeSystem/tide-code-system\|dataCoverage$ge100&_count=50 | 5 |
| Dominant frequency ≥ 8 Hz | Observation?component-code-value-quantity=http://example.org/tide/CodeSystem/tide-code-system\|dominantFrequency$ge8&_count=50 | 26 |
| Specific channel (Fp1) | Observation?bodysite=http://example.org/tide/CodeSystem/tide-channel-code-system\|Fp1&_count=50 | 3 |

The first three metric-level queries use the composite search parameter component-code-value-quantity, which ties a matched component code and value to the same component and avoids the false positives that two independently specified search parameters would produce. The channel-specific query uses bodySite, which is not a standard Observation search parameter in base FHIR R4. Therefore, we defined and registered a custom SearchParameter to enable it, consistent with the extensibility mechanism described in the Limitations and Future Work section.

All queries returned HTTP 200. The preliminary-status query returned exactly one result (sub-03 run-01), confirming that the status segmentation logic functions correctly. The subject-scoped query for sub-CUCOV003 returned 22 resources (one parent + 21 per-channel children), demonstrating that, because the subject reference is consistently populated across the parent and child Observations, both can be retrieved together via a single subject-based search, independent of any native server-side resolution of the hasMember relationship itself, which would otherwise require an explicit directive.

# Reference

1.	Sato M, Horiguchi I, Inoue M, Tomeoka K, Hatakeyama E, Kita Y, et al. EEG-Speech Brain Decoding Dataset. OpenNeuro. 2026. doi:10.18112/openneuro.ds007808.v1.0.0

2.	Calzada-Reyes A, Aubert-Vázquez E, Galán-García L, Bringas-Vega ML, Virués-Alba T, Charroó-Ruiz L, et al. A COVID-19 survivors and close contacts EEG dataset. OpenNeuro. 2026. doi:10.18112/openneuro.ds007823.v1.0.0

3.	Gramfort A, Luessi M, Larson E, Engemann DA, Strohmeier D, Brodbeck C, Goj R, Jas M, Brooks T, Parkkonen L, Hämäläinen M. MEG and EEG data analysis with MNE-Python. Front Neurosci 2013 Dec 26;7:267. PMID:24431986

4.	Harris CR, Millman KJ, Van Der Walt SJ, Gommers R, Virtanen P, Cournapeau D, Wieser E, Taylor J, Berg S, Smith NJ, Kern R, Picus M, Hoyer S, Van Kerkwijk MH, Brett M, Haldane A, Del Río JF, Wiebe M, Peterson P, Gérard-Marchant P, Sheppard K, Reddy T, Weckesser W, Abbasi H, Gohlke C, Oliphant TE. Array programming with NumPy. Nature 2020 Sept 17;585(7825):357–362. doi: 10.1038/s41586-020-2649-2

5.	Virtanen P, Gommers R, Oliphant TE, Haberland M, Reddy T, Cournapeau D, Burovski E, Peterson P, Weckesser W, Bright J, Van Der Walt SJ, Brett M, Wilson J, Millman KJ, Mayorov N, Nelson ARJ, Jones E, Kern R, Larson E, Carey CJ, Polat İ, Feng Y, Moore EW, VanderPlas J, Laxalde D, Perktold J, Cimrman R, Henriksen I, Quintero EA, Harris CR, Archibald AM, Ribeiro AH, Pedregosa F, Van Mulbregt P, SciPy 1.0 Contributors, Vijaykumar A, Bardelli AP, Rothberg A, Hilboll A, Kloeckner A, Scopatz A, Lee A, Rokem A, Woods CN, Fulton C, Masson C, Häggström C, Fitzgerald C, Nicholson DA, Hagen DR, Pasechnik DV, Olivetti E, Martin E, Wieser E, Silva F, Lenders F, Wilhelm F, Young G, Price GA, Ingold G-L, Allen GE, Lee GR, Audren H, Probst I, Dietrich JP, Silterra J, Webber JT, Slavič J, Nothman J, Buchner J, Kulick J, Schönberger JL, De Miranda Cardoso JV, Reimer J, Harrington J, Rodríguez JLC, Nunez-Iglesias J, Kuczynski J, Tritz K, Thoma M, Newville M, Kümmerer M, Bolingbroke M, Tartre M, Pak M, Smith NJ, Nowaczyk N, Shebanov N, Pavlyk O, Brodtkorb PA, Lee P, McGibbon RT, Feldbauer R, Lewis S, Tygier S, Sievert S, Vigna S, Peterson S, More S, Pudlik T, Oshima T, Pingel TJ, Robitaille TP, Spura T, Jones TR, Cera T, Leslie T, Zito T, Krauss T, Upadhyay U, Halchenko YO, Vázquez-Baeza Y. SciPy 1.0: fundamental algorithms for scientific computing in Python. Nat Methods 2020 Mar 2;17(3):261–272. doi: 10.1038/s41592-019-0686-2
