# Supplementary material for: Exchange of High-Frequency Medical Monitoring Data Using Fast Healthcare Interoperability Resources for Interoperable Integration Into Clinical Practice and Research: Methodological Development Study
Source: JMIR Med Inform. 2026 Nov 2;14:e94282. doi: 10.2196/94282 (PMC13727023; doi:10.2196/94282)
Supplement: Supplementary file 3 [file medinform-v14-e94282-s003.docx]

Practical comparison

Table M3.1 presents a structured methodological comparison of TIDE against three alternative FHIR-based approaches across key implementation dimensions.

**Table M3.1.** Overview of methodological comparison.

| **Comparison dimension** | **TIDE** | **UV PoCD SampleArrayObservation** | **SampledData** | **DocumentReference** |
| --- | --- | --- | --- | --- |
| **Raw-data linkage strategy** | Mandatory RawDataEndpoint extension referencing a dedicated TIDE Endpoint profile; storage backend fully decoupled from the Observation | Persistent linkage with provenance to source data | Embedded within the Observation resource as an encoded character string | Direct attachment or reference to external files (e.g., EDF) |
| **Multi-channel support** | Flat model: all channels as component slices of one Observation; hierarchical model: per-channel child Observations via hasMember | Explicit channel-level representation | Partial (multiple SampledData elements within one Observation) | Implicit (inside file payload) |
| **Terminology bindings** | TIDECodeSystem, TIDESupplementalCodes, TIDEChannelCodeSystem, TIDEConnectionTypeCodes, TIDEValueSet and  TIDEChannelValueSet ;  LOINC and SNOMED CT for modality codes | MDC / IEEE 11073 | UCUM units; no mandatory code binding | Optional coding |
| **Cardinality constraints** | Profile-governed: effectivePeriod 1..1, subject 1..1, RawDataEndpoint extension 1..1 | Explicit and profile-governed | Minimal — no mandatory slicing | Flexible and minimally constrained |
| **Provenance tracking** | Recording-segment level via effectivePeriod and status (preliminary/final); device-level via Device reference | Feature- and observation-level | Observation-level | Document-level |
| **Interoperability approach** | Semantic and statistical interoperability; metadata queryable without raw-data retrieval | Semantic interoperability | Semantic self-description; payload requires full parsing | File exchange |
| **Implementation complexity** | Moderate | Moderate | Low | Low |

DocumentReference follows a document-preservation strategy in which all data is the primary source of truth, and downstream consumers are responsible for extracting meaning from it. UV PoCD follows a knowledge-representation strategy in which high-frequency monitoring data characteristics are modeled explicitly, making them searchable, computable, and interoperable across systems. SampledData embeds signal values directly within the Observation resource as an encoded character string, enabling semantic self-description but requiring full deserialization prior to analysis. TIDE implements a Metadata-Proxy pattern, storing acquisition parameters and derived analytics as queryable FHIR component slices while offloading raw signal data to specialized external repositories via a mandatory Endpoint extension.

Table M3.2 compares signal quality and coverage between TIDE and three alternative FHIR-based approaches for high-frequency monitoring data, indicating which slices each approach can represent natively.

**Table M3.2.** Static comparison of the 16 TIDE component slices against the same alternative approaches.

| **Slice** | **Domain** | **TIDE** | **UV PoCD SampleArrayObservation** | **SampledData** | **DocumentReference** |
| --- | --- | --- | --- | --- | --- |
| **samplingFrequency** | Acquisition | **✓** | **✓** | **✓** | **✗** |
| **sampleCount** | Acquisition | **✓** | **✓** | **✓** | **✗** |
| **channelCount** | Acquisition | **✓** | **✓** | partial | **✗** |
| **signalMean** | Statistical summary | **✓** | **✗** | **✗** | **✗** |
| **signalMin** | Statistical summary | **✓** | **✗** | **✓** | **✗** |
| **signalMax** | Statistical summary | **✓** | **✗** | **✓** | **✗** |
| **signalStdDev** | Statistical summary | **✓** | **✗** | **✗** | **✗** |
| **anomalyCount** | Data quality | **✓** | **✗** | **✗** | **✗** |
| **eventCount** | Data quality | **✓** | partial | **✗** | **✗** |
| **dataCoverage** | Data quality | **✓** | **✗** | **✗** | **✗** |
| **dataGaps** | Data quality | **✓** | **✗** | **✗** | **✗** |
| **signalStabilityIndex** | Data quality | **✓** | **✗** | **✗** | **✗** |
| **samplingJitter** | Data quality | **✓** | **✗** | **✗** | **✗** |
| **dominantFrequency** | Data quality | **✓** | **✗** | **✗** | **✗** |
| **trendSlope** | Data quality | **✓** | **✗** | **✗** | **✗** |
| **signalEntropy** | Data quality | **✓** | **✗** | **✗** | **✗** |
| **Total supported** |  | **16/16 (100%)** | **3/16 (19%)** | **4/16 (25%)** | **0/16 (0%)** |

Legend: **✓**: fully supported ; **partial**: partially supported; **✗**: not supported

To complement the structural comparison with empirical evidence, we compared the bundle size required to represent identical input data across all four approaches. For each of the 5 source EDF recordings from the two demonstration datasets described in Multimedia Appendix 2, we extracted the same representative time window (5s for the 128-channel / 1200 Hz recordings, 60s for the 21-22-channel / 200 Hz recordings) and generated a FHIR transaction Bundle for each approach from the identical time window. Window lengths were chosen to maintain a comparable number of raw samples per file and ensure traceable bundle sizes. For this representative window, the resulting bundle size was 0.85 MB for TIDE, 19.18 MB for SampledData, 0.004 MB for DocumentReference, and 19.24 MB for UV PoCD SampleArrayObservation.

For TIDE and DocumentReference, payload size is independent of recording duration because they store a fixed number of computed metrics per channel, or a bare pointer, respectively. Therefore, the measured bundle size also represents the full duration. SampledData and PoCD embed raw samples directly, causing their payload size to scale linearly with sample count. Their full-duration sizes were therefore projected from the measured window using the ratio of full duration to window duration and summed across all 5 recordings (combined full duration ≈ 2.4 h). This is reported as a linear projection from the measured window, as reported in Table M3.3, and full-duration payloads were not directly ingested.

**Table M3.3.** Full-recording-duration projection (linear extrapolation)

| Approach | Scales with recording duration? | Bundle size at full recording duration (MB) |
| --- | --- | --- |
| TIDE | No; fixed-cardinality computed metrics per channel | 0.85 |
| SampledData | Yes; raw samples embedded in the resource | 8472.68 |
| DocumentReference | No, pointer to external file only | 0.004 |
| UV PoCD SampleArrayObservation | Yes, raw samples embedded per channel | 8505.31 |

These results are consistent with the structural comparison in Tables M3.1-M3.2. DocumentReference is the smallest of the four approaches because it stores only a pointer to the external file and performs no signal decomposition, which is an expected consequence of its document-preservation strategy, not a limitation specific to this benchmark.

TIDE's bundle size is roughly two orders of magnitude larger than DocumentReference’s pointer-only payload, while SampledData and PoCD are roughly 20x larger at the small representative window and scale linearly with recording duration, reaching an estimated four orders of magnitude larger than TIDE at the full duration of the tested recordings.

The more consequential difference, however, is structural rather than a matter of degree: TIDE is the only one of the four approaches for which a metric-level query (e.g., filtering by signalStabilityIndex via the composite search parameter component-code-value-quantity) can be expressed as a native FHIR search at all, because it is the only approach that stores computed, derived metrics in a structured, queryable field rather than only the raw signal.
